# Two new species of the genus *Comidoblemmus* Storozhenko & Paik from China (Orthoptera, Gryllidae)

**DOI:** 10.3897/zookeys.504.9232

**Published:** 2015-05-19

**Authors:** Haoyu Liu, Fuming Shi

**Affiliations:** 1 Museum, Hebei University, Baoding 071002, China; 2 College of Life Sciences, Hebei University, Baoding 071002, China

**Keywords:** Orthoptera, Gryllidae, *Comidoblemmus*, new species, China

## Abstract

Two new species of *Comidoblemmus* Storozhenko & Paik, 2009 are described and illustrated, *Comidoblemmus sororius* **sp. n.** (CHINA, Zhejiang) and *Comidoblemmus excavatus* **sp. n.** (CHINA, Guizhou). A key and a distribution map of all species in the world are presented.

## Introduction

The genus *Comidoblemmus* was established by Storozhenko and Paik (2009) for *Gryllus nipponensis* Shiraki, 1911, by monotypy and original designation. Except for the type species, which is widely distributed in Japan, Korea and China (Taiwan), none has been added to this genus until now (Eades et al. 2014).

**Map 1.**
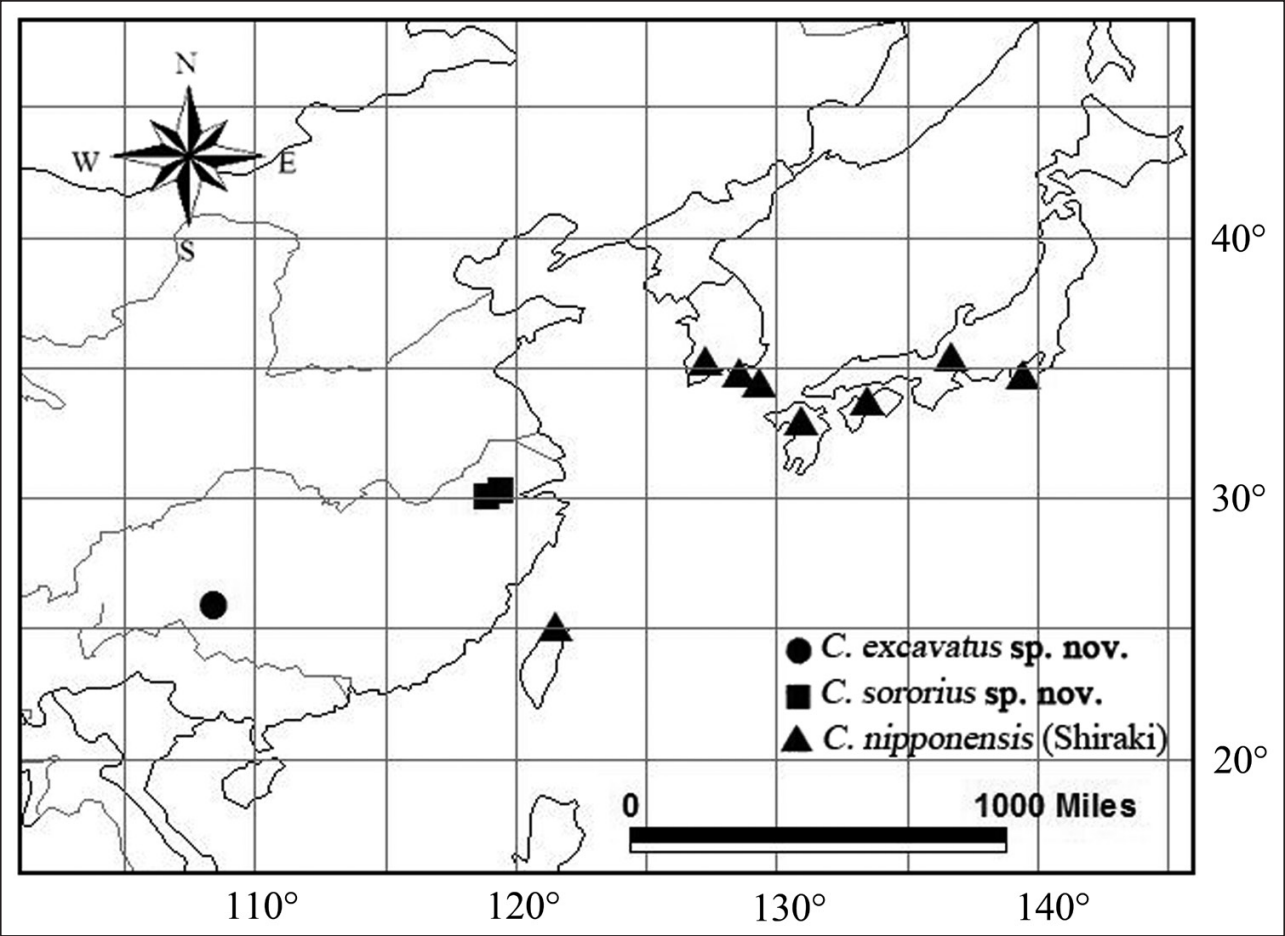
Distribution map of the genus *Comidoblemmus* Storozhenko & Paik.

During our study, two new species of *Comidoblemmus* from China were recently discovered and are described here under the names of *Comidoblemmus sororius* sp. n. and *Comidoblemmus excavatus* sp. n. They match the generic diagnosis well and are characterized by having four pairs of dorsal spines on each hind tibia and similar shapes of male genitalia as the type species, epiphallus with posterior margin between lateral lobes rounded, not sinuate. But they are different from *Comidoblemmus nipponensis* (Shiraki, 1911) by the distinctly oblique head, whose shape could be more or less variable owing to the agonistic behavior character (Storozhenko and Paik 2009) within one genus, such as *Loxoblemmus* Saussure, 1877 (sensu Gorochov 2001). Thus, we confirm the two new species belong to the genus *Comidoblemmus*, which currently includes three species. A key for their identification and a distribution map (Map 1) are presented.

## Material and methods

The type specimens of the new species are deposited in the Museum, Hebei University, Baoding, China (MHBU).

The male genitalia were dissected and cleared in 10% KOH solution. All morphological structures were photographed using a Leica M205A microscope. Images of multiple layers were stacked using Combine ZM. Distribution maps were prepared using the geographic information system software ArcView 3.2 (ESRI, Redlands, CA, USA), based on localities of the specimens examined for this study and those mentioned in the literature (Shiraki 1911, 1930, Chopard 1961, Randell 1964, Ichikawa et al. 2000, Ichikawa et al. 2006, Storozhenko and Paik 2007, 2009).

## Taxonomy

### Key to the species of *Comidoblemmus* Storozhenko & Paik

**Table:** 

1	Head with both genae nearly parallel in frontal view (Storozhenko and Paik 2009: Fig. 1); posterior margin between lateral lobes of epiphallus almost straight (Storozhenko and Paik 2009: Figs 10–12)	***Comidoblemmus nipponensis* (Shiraki, 1911)**
–	Head with genae distinctly converging downwards in frontal view (Figs 3, 4, 7); posterior margin between lateral lobes of epiphallus distinctly rounded	**2**
2	Male tegmina reaching abdominal apex (Fig. 1); male supra anal plate slightly narrowed posteriorly, posterior margin narrowly rounded (Figs 9–11)	***Comidoblemmus sororius* sp. n.**
–	Male tegmina reaching 8^th^ abdominal tergite (Fig. 6); male supra anal plate distinctly narrowed posteriorly, posterior margin slightly emarginated in middle (Figs 12–14)	***Comidoblemmus excavatus* sp. n.**

**Figures 1–8.**
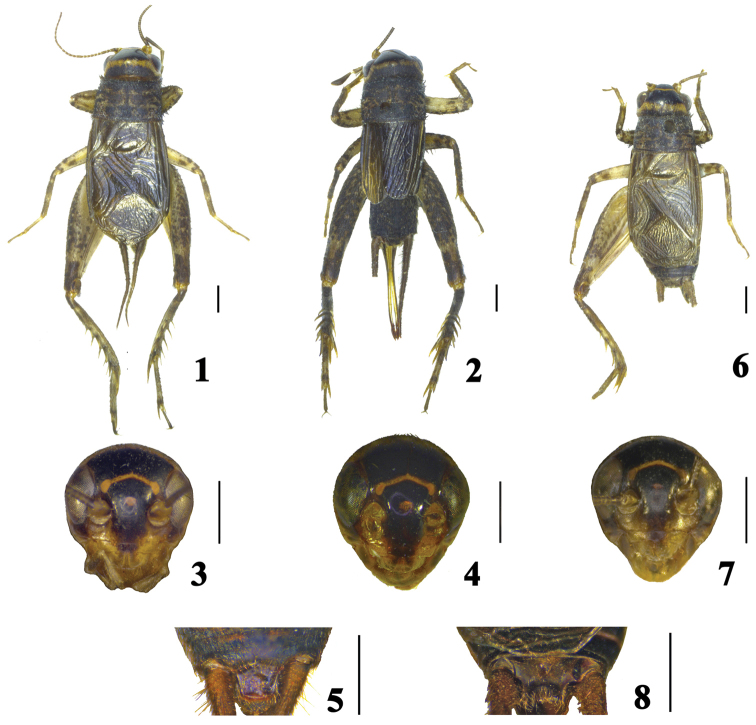
*Comidoblemmus* spp. **1–5** *Comidoblemmus sororius* sp. n. (**1, 3, 5** male; **2, 4** female) **6–8** *Comidoblemmus excavatus* sp. n. (male): **1, 2, 6** habitus, dorsal view **3, 4, 7** head, frontal view; **5, 8** supra anal plate, dorsal view. Scale bars: 1.0 mm.

**Figures 9–14.**
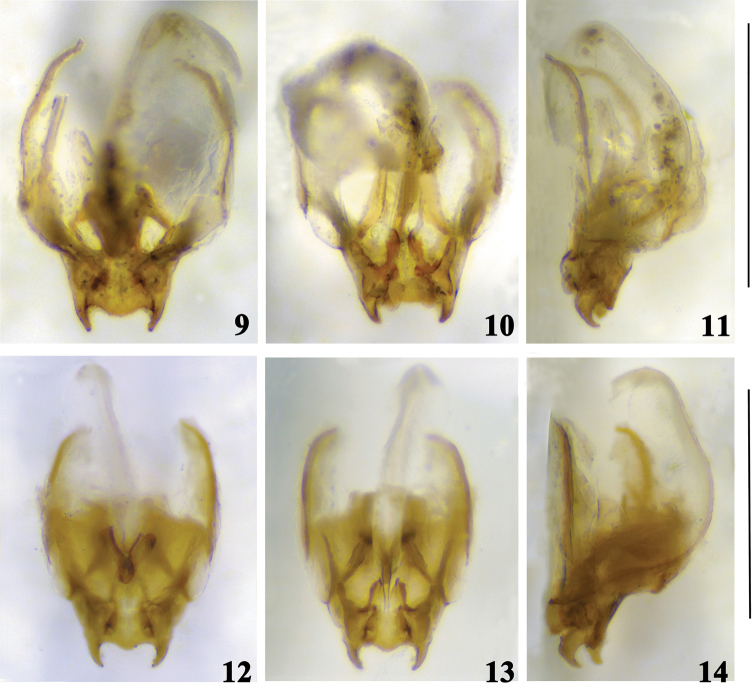
Male genitalia of *Comidoblemmus* spp. **9–11** *Comidoblemmus sororius* sp. n. **12–14** *Comidoblemmus excavatus* sp. n.: **9, 12** dorsal view **10, 13** ventral view **11, 14** lateral view. Scale bars: 1.0 mm.

### Comidoblemmus sororius sp. n.

Taxon classificationAnimaliaOrthopteraGryllidae

http://zoobank.org/ABF68F2D-C329-440F-BA69-755D1163146F

Figs 1–5 , 9–11

#### Type material.

Holotype ♂: CHINA: Zhejiang, Lin’an, Tianmushan, Qianmutian, 14.–15.IX.2012, leg. Y.Y. Lu. Paratypes: 9♂♂, 1♀: same data as the holotype; 2♀♀: Zhejiang, Lin’an, Qingliangfeng, Shunxiwu, 17.–19. IX.2012, leg. Y.Y. Lu.

#### Description.

Male (Fig. 1). Body small-sized. Head nearly globular (Fig. 3), slightly wider than anterior margin of pronotum, frontal rostrum short and about 1.8 times as wide as scapus; eyes large, oval; last joint of maxillary palpus slightly longer than 4^th^ joint, distinctly widened apicad. Pronotum transverse, slightly widened posterioly, about 0.6 times as long as width of posterior margin, anterior and posterior margins straight. Tegmina reaching abdominal apex, present with 3 oblique veins, mirror large, about 1.2 times as long as wide, apical field very short; wings absent. Fore tibia with two tympana, outer tympanum large and oblong, about 2.8 times as long as wide, inner tympanum small and nearly round. Hind femur slender, about 2.9 times as long as maximal width; hind tibia with 4 pairs of dorsal spines and 3 pairs of apical spurs, of which middle one longest while lower one shortest among the inner spurs, lower and upper ones equal in length and distinctly shorter than middle one among outer spurs; first hind tarsal segment each side with 4–5 small spines in a line on dorsal surface.

Supra anal plate (Fig. 5) slightly narrowed posteriorly, with posterior margin narrow and rounded at apex. Subgenital plate rather long, narrowed posteriorly, nearly coniform. Genitalia (Figs 9–11): epiphallus with two lateral lobes on posterior margin, and posterior margin between lateral lobes of epiphallus distinctly widely rounded; lateral lobes acute at apex and bent upwards apicad in lateral view; ectoparamers small.

Female (Fig. 2). Head very similar to that of male (Fig. 4). Lateral margins of pronotum nearly parallel. Tegmina reaching 5^th^ abdominal tergite, present with several parallel veins. Ovipositor straight, spear-shaped, 1.2 times shorter than hind femur.

Coloration. Body black brown. Head black, with a narrow transverse yellow stripe in middle of dorsum and between lateral ocelli respectively, mouthparts light yellow. Pronotum black, disc with light yellow markings. Legs yellowish brown mixed with irregular dark brown markings, hind femur with numerous oblique black markings on outer surface. Ovipositor brown.

#### Measurements

(mm). Male: body 7.0–8.1, pronotum 1.4–1.5, tegmen 4.6–5.0, hind femur 5.2–5.7; female: body 6.9–8.0, pronotum 1.4–1.5, tegmen 3.2–3.5, hind femur 5.4–5.9, ovipositor 4.2–4.5.

#### Diagnosis.

This new species is similar to *Comidoblemmus nipponensis* (Shiraki), but differs from the latter by the male tegmina reaching abdominal apex; head with genae distinctly converging downwards in frontal view; posterior margin of supra anal plate narrowly rounded; posterior margin between lateral lobes of epiphallus distinctly widely rounded.

#### Distribution.

China (Zhejiang).

#### Etymology.

The specific name is derived from Latin *soror* (sisters), referring to this species is similar to *Comidoblemmus nipponensis* (Shiraki).

### Comidoblemmus excavatus sp. n.

Taxon classificationAnimaliaOrthopteraGryllidae

http://zoobank.org/5A43343F-DAF4-492C-9056-B653A8D24EE1

Figs 6–8 , 12–14

#### Type material.

Holotype ♂: CHINA: Guizhou, Leishan, Fangxiang, 15.IX.2005, leg. H.Y. Liu.

#### Description.

Male (Fig. 6). Body small-sized. Head nearly globular (Fig. 7), slightly wider than anterior margin of pronotum, frontal rostrum short and about 1.4 times as wide as scapus; eyes large, oval; last joint of maxillary palpus slightly longer than 4^th^ joint, distinctly widened apicad. Pronotum transverse, slightly widened posterioly, about 0.6 times as long as width of posterior margin, anterior and posterior margins straight. Tegmina reaching 8^th^ abdominal tergite, present with 3 oblique veins, mirror large, about 1.4 times as long as wide, apical field short; wings absent. Fore tibia with two tympana, outer tympanum large and oblong, about 2.9 times as long as wide, inner tympanum small and nearly round. Hind femur slender, about 2.8 times as long as maximal width; hind tibia with 4 pairs of dorsal spines and 3 pairs of apical spurs, of which middle one longest while lower one shortest among the inner spurs, lower and upper ones equal in length and distinctly shorter than middle one among outer spurs; hind first tarsal segment each side with 5 small spines in a line on dorsal surface.

Supra anal plate (Fig. 8) distinctly narrowed posteriorly, with posterior margin slightly emarginated in middle. Subgenital plate rather long, narrowed posteriorly, nearly coniform. Genitalia (Figs 12–14): epiphallus with two lateral lobes on posterior margin, and posterior margin between lateral lobes of epiphallus distinctly narrowly rounded; lateral lobes acute at apex and bent upwards apicad in lateral view; ectoparamers small.

Female. Unknown.

Coloration. Body black brown. Head black, with a narrow transverse yellow stripe in middle of dorsum and between lateral ocelli respectively, mouthparts light yellow. Pronotum black, disc with light yellow markings. Tegmina brown. Legs yellowish brown with irregular dark brown markings, and hind femur with numerous oblique black markings on outer surface.

#### Measurements

(mm). Male: body 8.8, pronotum 1.7, tegmen 6.0, hind femur 5.8.

#### Diagnosis.

This new species is similar to *Comidoblemmus nipponensis* (Shiraki), but differs from the latter by the head with checks distinctly converging downwards in frontal view; posterior margin of supra anal plate slightly emarginated in middle; posterior margin between lateral lobes of epiphallus distinctly narrowly rounded. It also resembles *Comidoblemmus sororius* sp. n., but can be distinguished by the posterior margin of supra anal plate slightly emarginated in middle; posterior margin between lateral lobes of epiphallus distinctly narrowly rounded; tegmina reaching 8^th^ abdominal tergite, mirror distinctly longer than wide.

#### Distribution.

China (Guizhou).

#### Etymology.

The specific name is derived from Latin *ex*- (out) + *cavare* (cave), referring to its posterior margin of supra anal plate slightly emarginated in middle.

## Supplementary Material

XML Treatment for Comidoblemmus sororius

XML Treatment for Comidoblemmus excavatus

## References

[B1] Chopard L (1961) Les divisions du genre *Gryllus* basées sur l’ étude de l’appareil copulateur (Orth. Gryllidae). Eos, Revista Espanola de Entomologia 37: 267–287.

[B2] Eades DC Otte D Cigliano MM Braun H (2014) Orthoptera Species File. Version 5.0/5.0. http://Orthoptera.SpeciesFile.org [accessed 22–December–2014]

[B3] Gorochov AV (2001) Remarkable examples of convergence and new taxa of Gryllini (Orthoptera: Gryllidae). Zoosystematica Rossia 9(2): 316–350.

[B4] Ichikawa A Ito F Kano Y Kawai M Tominaga I Murai T (2006) Orthoptera of the Japanese Archipelago in Color. Hokkaido University Press, Sapporo, 687 pp [In Japanese]

[B5] Ichikawa A Murai T Honda E (2000) Monograph of Japanese crickets (Orthoptera: Grylloidea). Bulletin of the Hoshizaki Green Founddation 4: 257–332. [In Japanese]

[B6] Randell RL (1964) The male genitalia in Gryllinae (Orthoptera: Gryllidae) and a tribal revision. Canadian Entomologist 96(12): 1565–1607. doi: 10.4039/Ent961565-12

[B7] Shiraki T (1911) Monographie der Grylliden von Formosa, mit der Übersicht der Japanischen Arten. Generalgouvernment von Formosa, Taihoku, 129 pp.

[B8] Shiraki T (1930) Orthoptera of the Japanese Empire. Part I. (Gryllotalpidae and Gryllidae). Insecta Matsumurana 4(4): 181–252.

[B9] Storozhenko SYu Paik JCh (2007) Orthoptera of Korea. Dalnauka, Vladivostok, 232 pp.

[B10] Storozhenko SYu Paik JCh (2009) A new genus of cricket (Orthoptera: Gryllidae: Gryllinae) from East Asia. Zootaxa 2017: 61–64.

